# Uncommon Occurrences of Air Embolism: Description of Cases and Review of the Literature

**DOI:** 10.1155/2018/5808390

**Published:** 2018-07-08

**Authors:** Giorgio Berlot, Adriano Rinaldi, Marco Moscheni, Massimo Ferluga, Perla Rossini

**Affiliations:** Anesthesia and Intensive Care Department, Cattinara Hospital, Azienda Sanitaria Universitaria Integrata di Trieste, University of Trieste, Strada di Fiume 447, 34149 Trieste, Italy

## Abstract

Many different risk factors have been associated with the occurrence of gas embolism making this potentially lethal complication easily avoidable. However, this condition can occur in circumstances not commonly reported. Three different and extremely uncommon cases of gas embolism are presented and discussed: the first was caused by the voluntary ingestion of hydrogen peroxide, the second occurred during a retrograde cholangiopancreatography, and the last followed the intrapleural injection of Urokinase. Whereas in the first patient the gas embolism was associated with only relatively mild digestive symptoms, in the remaining two it caused a massive cerebral ischemia and an extended myocardial infarction, respectively. Despite a hyperbaric oxygen therapy performed timely in each case, only the first patient survived. The classical risk factors associated with gas embolism like indwelling central venous catheters, diving accidents, etc. are rather well known and thus somewhat preventable; however, a number of less common and difficult-to-recognize causes can determine this condition, making the correct diagnosis elusive and delaying the hyperbaric oxygen therapy, whose window of opportunity is rather narrow. Thus, a gas embolism should be suspected in the presence of not otherwise explainable sudden neurologic and/or cardiovascular symptoms also in circumstances not typically considered at risk.

## 1. Introduction

The term gas embolism (GE) indicates an array of clinical conditions determined by the entry of gases like air, oxygen (O_2_), carbon dioxide (CO_2_), or nitrogen (N), and so on, into the bloodstream driven by the pressure gradient existing between the atmosphere and the venous system [1]. The severity of the clinical picture basically depends on some gas-related factors, including the body's position, the blood solubility, the patient's overall health, the volume, and the rate of accumulation [2, 3]; indeed, even small amounts can determine severe clinical consequences if located in critical locations: in humans, the injection of as few as 1 to 2 ml of air in the arterial circulation has been associated with neurologic damage and extremely severe consequences, even cardiac arrest, have been described after the injection of 2 ml only of air into the cerebral circulation [4–8]. In the past few decades, a better knowledge of the pathophysiology of GE determined an increased awareness of the underlying risk factors and clinical manifestations and prompted the publication of a number of clinical and experimental studies [7, 9–13].

Here we report and discuss three cases of GE determined by unusual causes, including the voluntary ingestion of hydrogen peroxide (HP), an endoscopic procedure, and the Urokinase instillation adjunctive to the chest tube drainage in a case of pleural empyema.

## 2. Cases Report

### 2.1. Case Number 1

**Figure 1:**
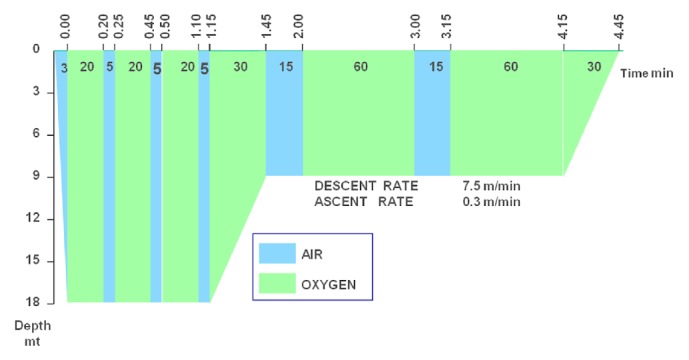
US Navy decompression Table 6.

**Figure 2:**
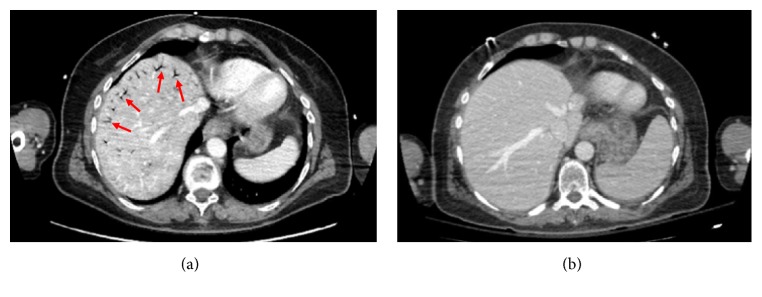
Abdominal CT scan: (a) GE in the peripheral branch of the portal system after HP ingestion; (b) reduction of the GE after HBOT.

A 60-year-old woman was transferred from another hospital after the voluntary ingestion of 400 ml of 5% hydrogen peroxide (HP). At the admission, she was awake and complained of mild diffuse abdominal pain; on examination, the abdomen appeared slightly distended and the bowel sounds were increased. Once in our ICU, the HBOT started 7 hours after the ingestion and was performed according to the US Navy Table 6 (Figure 1). A pre-HBOT abdominal CT scan demonstrated multiple gas bubbles distributed in the peripheral branches of the portal system and in the lumen of the upper mesenteric, ileocolic, and colic veins (Figure 2(a)), which appeared reduced after the treatment (Figure 2(b)). On the following day, a TEE demonstrated a PFO. Three days later she was discharged home free of symptoms.

### 2.2. Case Number 2

**Figure 3:**
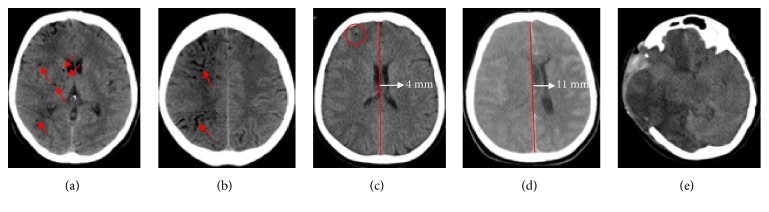
Head CT scan: (a) before HBOT: gas bubbles in the basal nuclei and (b) within the subarachnoid spaces (arrows); (c) immediately after HBOT: single right frontal bubble (circle) and 4 mm: leftward deviation of the midline (arrow); (d) 14 hours after HBOT 4 mm: leftward deviation of the midline; (e) 5 days after right decompressive craniectomy: massive brain herniation with right hemispheric and left frontal ischemia.

A 62-year-old woman underwent an endoscopic retrograde cholangiography (ERCP) using air to dilate the duodenum in another hospital. During the procedure, performed under light iv. sedation, the patient suddenly presented seizures, spontaneous decerebrate posture, bilateral mydriasis, associated with atrial fibrillation, and arterial desaturation; at this point she was tracheally intubated and mechanically ventilated. A CT scan of the head demonstrated many small gas bubbles within the subarachnoid spaces, the intrasellar space, and the basal nuclei (Figures 3(a) and 3(b)). The patient was taken immediately to our HBOT facility; the treatment started 4 hours and 45 minutes after the onset of symptoms and followed the US Navy Table 6 (Figure 1). A CT scan obtained immediately thereafter showed the reduction of the subarachnoid air and a single right frontal bubble; there was an effacement of the right hemispheric sulci and a 4 mm-left bound deviation of the midline (Figure 3(c)). Mannitol and hypertonic saline were given to decrease the intracranial pressure (ICP) and the sedation was maintained with iv. Thiopentone. On the next morning, at the CT scan of the brain the midline shift was increased up to 11 mm and the basal cisternae were not visible (Figure 3(d)). At the suspension of the sedation, the patient presented right myoclonus, bilateral left deviation of the eyes and left hyperextension at the painful stimulation. A wide right craniotomy was then performed to reduce the ICP but the brain herniated through the cranial breach (Figure 3(e)). The patient returned to the initial hospital three days later and died in vegetative state seven months after the described episode.

### 2.3. Case Number 3

**Figure 4:**
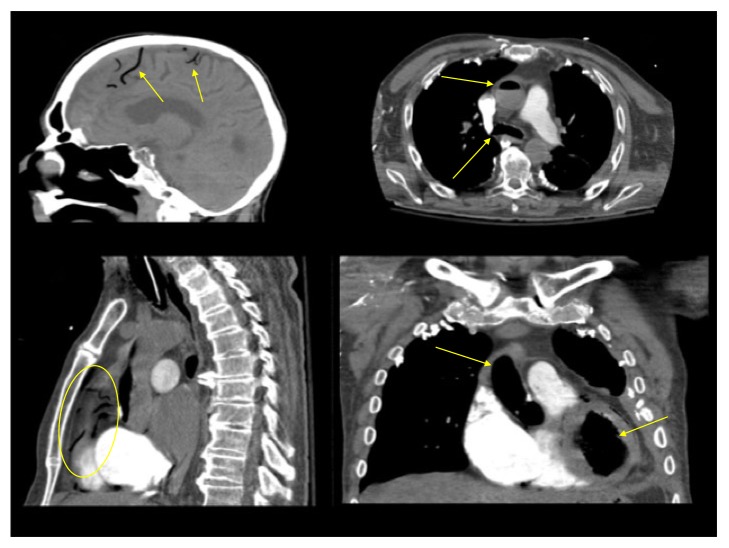
(a): Air within the cortical vessels (arrows); (b) air within the ascending and thoracic descending aorta (arrows); (c) air within the coronary arteries (circle); (d) air within the aortic arch and the left ventricle.

**Figure 5:**
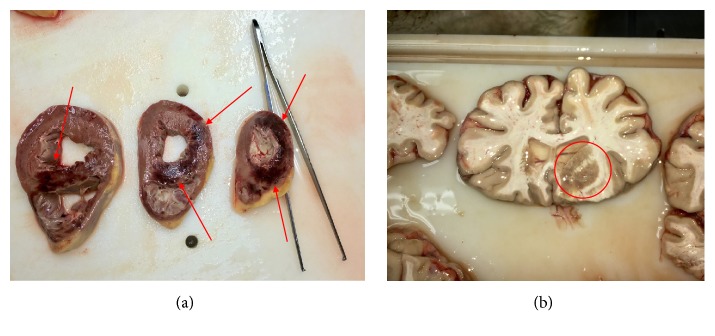
(a) Acute myocardial infarction involving extensively the right ventricle and the septum; (b) acute cerebral infarction downstream the arteries obstructed by the GE.

A 72-year-old male patient underwent a left thoracostomy for the positioning of a chest tube to treat an empyema complicating a mesothelioma. On the day after, Urokinase was injected through the chest tube, which was clamped immediately thereafter. Approximately 10 minutes later, the patient suddenly became unconscious and cyanotic. At the arrival of the medical emergency team, the patient was comatose, anisocoric, bradycardic, and hypotensive. The patient was intubated and mechanically ventilated at FIO_2_ 100%; at the same time, fluids, dobutamine, and norepinephrine were started. The CT scan of the chest and of the brain demonstrated a massive GE with evidence of multiple air bubbles inside the thoracic aorta, the left cardiac ventricle, the coronary arteries, and the cortical vessels (Figure 4). The HBOT started three hours after the onset of symptoms and followed the US Navy Table 6 (Figure 1). During the treatment the patient underwent repeated episodes of ventricular tachycardia and fibrillation treated according to the BLS guidelines and for this reason it has not been possible to follow the US Navy Table 6A, which was our first choice; thirty minutes before the end of the procedure an asystole appeared and the CPR was stopped 20 minutes after its occurrence. The autopsy failed to demonstrate any laceration of the lung, whose surface presented multiple wide hemorrhagic areas; the cause of the cardiogenic shock was ascribed to a hyper acute extensive myocardial infarction; a wide ischemic area was present also in the right frontal lobe (Figure 5).

## 3. Discussion

**Figure 6:**
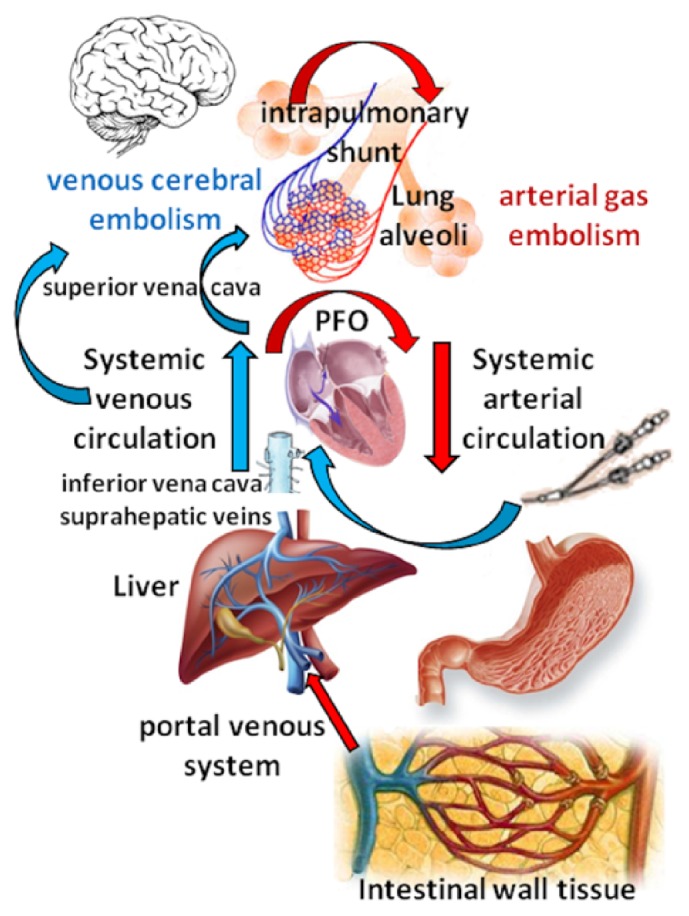
Routes of dissemination of GE.

Several different pathologic and nonpathologic conditions have been associated with the occurrence of GE, including thoracic penetrating injuries, mechanical ventilation with elevated pressures of insufflations, surgical procedures performed in the sitting position, endoscopic maneuvers, air aspiration from central venous catheters (CVC), or their subcutaneous tracks, sexual intercourses, diving accidents, etc. [14]. Whatever the cause, after the penetration in the venous system the gas bubbles can travel along different routes (Figure 6): first, they can join the venae cavae and move upward into the internal jugular vein (IJV) due to both its continuity with the upper vena cava and its shorter length; GE in this location impede the cerebral venous drainage. Second, they can reach the lung circulation and obstruct the right ventricular outflow. Finally, they can migrate towards the systemic circulation via physiologic intrapulmonary shunts, the pulmonary capillaries, or a patent foramen ovale (PFO). Once in the aorta, the GE can move and plug the microvascular network everywhere, determining a multisystem disorder whose features are primarily related to either the ischemia or the subsequent inflammatory reaction of the involved organs [15]. Then, it appears that the GE-related symptoms vary according to their location, those determined by arterial GE (AGE) being more severe and sudden than those caused by venous GE (VGE), provided that the latter do not exert cardiovascular or neurologic effects.

The incidence of GE in different studies ranges from 1 in 772 to 2,65 per 100.000 hospitalizations [1]; this extremely wide difference is probably due to the lack of specificity of symptoms and/or the diagnostic tools used. Actually, as more and more invasive diagnostic and therapeutic procedures requiring the cannulation of large veins and/or the insufflation of gas inside the body cavities are performed, it is hypothesizable that an elevated number of GE do not cause symptoms or go unnoticed [1]. Hence, the diagnosis of GE is based primarily on the clinical suspicion, on presence of risk factors, and on the use of monitoring techniques that are able to detect air bubbles floating into the bloodstream, including the precordial Doppler ultrasound probe and the transesophageal echocardiography (TEE); the final diagnosis and the severity of the involvement of the target organs are established with the CT scan [1, 2].

The treatment of serious forms of GE warrants an aggressive approach, consisting in (a) the endotracheal intubation and mechanical ventilation with 100% O_2_; (b) the positioning of the patient in the Trendelenburg and left lateral decubitus to reduce the amount of GE floating to the brain and facilitate the egress of air from the right ventricle; (c) the aspiration of bubble from the right ventricle via a CVC; and (d), most importantly, the Hyperbaric Oxygen Therapy (HBOT), to be performed on an emergency basis to reduce the volume of the GE. Its rationale is based on sound chemicophysical principles, including the reduction of the volume of the bubbles (Boyle's law) and the increased solubility of the culprit gas (Henry's law) leading to a better oxygenation of the ischemic tissues; moreover, HBOT has been associated with the reduction of the inflammatory reaction in the ischemic areas by limiting the activation and the adhesion of the leukocytes upon the endothelium [16–18]. The window of opportunity of HBOT is relatively narrow, as demonstrated by Blanc [19] who reported a better outcome in patients with cerebral GE treated < 6 hours after the event as compared with those treated after this interval; however, patients with GE can gain advantage from a HBOT even after a significant delay [20–22]. Even if quite often the source of GE is immediately clear or easily detectable, there are many variables, including the rarity of reported cases, the presence of confounding factors, and the failed recognition of triggering events which can make the diagnosis extremely elusive and delay if not impede the appropriate treatment.

In ICU patients, the diagnosis of GE can be relatively straightforward as a limited number of causes can prompt its occurrence; conversely, far more difficulties are present in non-ICU patients due to uncommon triggering events like those exposed above.

This is well represented by the first patient, who ingested a consistent amount of HP. This substance, which has multiple uses including antisepsis, is hydrolyzed by the enzyme catalase via the reaction: 2H_2_O_2_ → 2 H_2_O + O_2_ + heat; the amount of released O_2_ is related to both the amount and the concentration of HP: as an example, one milliliter of 6% HP releases approximately 10 mL of O_2_ [23]. The ingestion of HP can exert deleterious effects via different mechanisms, including (a) the direct cytotoxic action on the gastric mucosa possibly leading to erosions and perforations; (b) the increase of the intragastric volume and pressure determined by the high amounts of O_2_ derived by its hydrolysis; and (c) the absorption of O_2_bubbles through the gastroduodenal wall and its passage into the portal system where it can determine portal hypertension; moreover, the gas bubbles can migrate from the liver sinusoids or preexisting portocaval shunts into the inferior vena thus reducing the venous return [24, 25]. Our patient did not present these major complications despite the relevant volume of highly concentrated HP ingested, making the choice of treatment uncertain. Actually, the role of HBOT in asymptomatic patients is not clear, as some patients treated conservatively were discharged without consequences [23, 26], probably due to a more rapid scavenging of O_2_ bubbles as compared with those containing N only as it occurs in diving accidents [23]; thus, the HBOT was performed more to prevent a systemic embolization (a PFO which was demonstrated in the following days) than to reduce the volume of gas bubbles located in the portal circulation, which often dissolve spontaneously [27]. An immediate treatment is not warranted in case of a VGA confined to the portal system; actually, the patient improved and her abdominal symptoms resolved completely even if the HBOT was initiated after as long as 7 hours from the ingestion of HP.

The second patient suffered from the most uncommon complication of ERCP, consisting in the penetration of gas into the portal vein [28]. Different risk factors have been identified, including preexisting conditions, such as inflammation, previous surgeries, or a stone-induced decubitus of the biliary tract, and procedure-related factors, such as the irritation caused by the contrast medium, the sphincterotomy, the placement of a metal stent, and elevated volumes of air insufflated at pressure to distend the gastroduodenal tract [29, 30]. In our patient, this latter mechanism likely determined the intramural dissection of small veins and the subsequent passage of air bubbles into the suprahepatic veins through the hepatic sinusoids or via preexisting shunts between the portal and inferior cava vein [31]. The severe neurologic symptoms and their abrupt onset must be ascribed either to the concurrent presence of a retrograde VGE in the right IJV, and/or to air bubbles escaping into the systemic circulation through a intrapulmonary shunt or the transcapillary route that determined a massive ischemic injury in the territory of the right middle cerebral artery [30, 32, 33]. The diagnosis of cerebral GE was particularly difficult due to a number of circumstances, including the sedation, the lack of preliminary symptoms, and, most importantly, the extremely uncommon occurrence of this complication [29, 34, 35]. Despite the aggressive and multimodal treatment of this patient, which included also a decompressive craniotomy besides HBOT, the ischemic damage was so relevant that the neurologic conditions failed to improve in the following months.

The third case is somewhat puzzling, as the patient died in refractory cardiogenic shock likely due to the intracoronary AGE in the absence of any port of entry for air discovered at the autopsy. Actually, other authors have described the occurrence of GE in patients undergoing one or even repeated pleural lavages with fibrinolytic agents, sometimes after several days from the positioning of the chest tube. In our patient, we hypothesized that the injected Urokinase lysed one or more clots on the surface of the hemorrhagic areas demonstrated at the autopsy thus permitting the gas contained into the empyema to penetrate the pulmonary vessels and to cause a GE involving multiple vascular beds.

In all our patients the HBOT was performed according US Navy Table 6 which is currently used to treat patients involved in diving accident.

When discussing GE the key question is if it could have been somewhat prevented or recognized in an early phase as the treatment is time-dependent and the HBOT facilities can be difficult to reach due to technical factors, including weather or altitude limitations for helicopters and traffic jam for surface transportation. In the described cases, the prevention of GE clearly was not feasible in case number 1 but it could have been avoided using CO_2_ to inflate the duodenum in the second case. In patient number 3, this issue is difficult to establish, as other cases have been described in patients undergoing lung or pleural biopsies under thoracoscopy [36, 37]. In these circumstances, a high index of suspicion is warranted to detect as soon as possible neurologic and/or cardiovascular abnormalities possibly caused by a GE and to shorten the treatment-free interval.

## 4. Conclusions

Gas embolism can occur in circumstances other than those classically considered at risk thus making the diagnosis elusive. Any sudden change in mental status and/or hemodynamic alterations not otherwise explainable should raise the suspicion of GE during even minimally invasive procedures that can create a communication between air or other gases and the vascular system. If the suspicion of AGE is confirmed and there are no contraindications—like untreated pneumothorax—it is of primary importance to start the HBOT, which is a very safe medical procedure if it is done properly, as soon as possible in order to improve the correction of ischemia and the outcome of the patient.

You should also consider the possibility of HBOT treatments once a day for 90 minutes with a FiO_2_ 100% at 2 ATA if there are sequels after the main treatment.

## Data Availability

All data and materials, including CT scans and lab results, are available and can be provided if requested.
